# Cost analysis of glatiramer acetate versus interferon-β for relapsing-remitting multiple sclerosis in patients with spasticity: the Escala study

**DOI:** 10.1186/s13561-015-0066-2

**Published:** 2015-10-16

**Authors:** Rainel Sánchez-de la Rosa, Laura García-Bujalance, José Meca-Lallana

**Affiliations:** 1 Medical Department, TEVA Pharmaceutical, Calle de Anabel Segura, 11, 28108 Madrid, Spain; 2 Market Access & Regulatory Department, TEVA Pharmaceutical, Calle de Anabel Segura, 11, 28108 Madrid, Spain; 3 Neurology Department, Hospital Clínico Universitario Virgen de la Arrixaca, Carretera Madrid-Cartagena, S/N, 30120 El Palmar, Spain

**Keywords:** Cost analysis, Glatiramer acetate, Interferon-β, Relapsing-remitting multiple sclerosis, Spasticity

## Abstract

**Objective:**

The Escala Study evidenced that the administration of glatiramer acetate for relapsing-remitting multiple sclerosis improved the spasticity of patients previously treated with interferon-β. However, whether such an improvement was translated into cost savings remained unclear. We therefore conducted a cost analysis of glatiramer acetate versus interferon-β in these patients with multiple sclerosis and spasticity.

**Methods:**

This cost analysis encompassed data from the observational Escala Study, which included patients with relapsing-remitting multiple sclerosis and spasticity whose treatment had been switched from interferon-β to glatiramer acetate. Costs prior to starting glatiramer acetate (interferon-β period) were compared to the subsequent six months on glatiramer acetate (glatiramer acetate period). The analysis was carried out following the recommendations for conducting pharmacoeconomic studies and from the Spanish National Health System perspective. Costs associated with multiple sclerosis treatment, spasticity treatment and relapse management were expressed in 2014 euros (€); a 7.5 % discount was applied—when needed—as stipulated in Spanish law.

**Results:**

The management of relapsing-remitting multiple sclerosis, spasticity and relapses accounted for a 6-month cost per patient of 7,078.02€ when using interferon-β and 4,671.31€ when using glatiramer acetate. Switching from interferon-β to glatiramer acetate therefore represented a cost saving of 2,406.72€ per patient in favour of glatiramer acetate, which resulted from savings in treatment costs, relapse management and spasticity treatment of 1,890.02€, 430.48€ and 86.21€, respectively. The ratio of the costs during interferon-β was 1.5 times the costs during glatiramer acetate; thus, a fixed budget of 5,000,000€ would enable 1,070 patients to be treated with glatiramer acetate and only 706 patients with interferon-β.

**Conclusions:**

The treatment of relapsing-remitting multiple sclerosis with glatiramer acetate entailed cost savings when compared to interferon-β in patients with spasticity, which not only resulted from its lower costs of therapy and relapse management but also from its favourable effect on reducing spasticity. Thus, glatiramer acetate may be regarded as a more efficient alternative than interferon-β from the perspective of the Spanish National Health System.

## Background

Multiple sclerosis (MS) is a chronic, progressive, neurodegerenative, auto-immune and disabling disease of the central nervous system. Its most commonly diagnosed type is relapsing-remitting multiple sclerosis (RRMS), which is associated with a repeating pattern of relapses followed by a period of remission [1]. MS is the most common cause of neurological disability in young adults worldwide [2], with a widely variable prevalence ranging from 50 to 125 cases per 100,000 people/year in Spain [3–6]. MS has a considerable impact on patients’ quality of life, particularly during relapses and as the disease progresses [7]. It additionally represents a large economic burden for the patients, their careers, and the health service [8].

Drug therapies that are able to reduce the number of relapses and/or slow disease progression in RRMS are known as disease-modifying therapies (DMTs); a DMT is typically initiated as soon as a diagnosis of RRMS is confirmed. First-line basic therapy is distinguished from second-line escalation therapies. Usually, the treatment is started with basic therapeutics and the patient is monitored clinically with subsequent magnetic resonance imaging (MRI). In case of stable disease and well-tolerated treatment, the therapy is continued. In case of ongoing clinical and/or radiological disease activity and/ or relevant side effects, an escalation therapy can be initiated. In general, patients with ≥1 relapse per year, absence of complete recovery from relapses, sustained disability progression of ≥1 and MRI progression, with or without clinical signs, are considered as ‘treatment non-responders’, justifying the transition from basic to escalation therapy [9]. DMTs are basic therapeutics that include interferon-β (Avonex®, Betaferon®/Betaseron® and Rebif®) and glatiramer acetate (Copaxone®), which are immunomodulators used as first-line therapeutics with more than 20 years of experience. They reduce the annualized relapse rate by approximately 30 % and do not cause severe side effects [10]. At this time, there are three more oral drugs available: teriflunomide (Aubagio®), dimetil fumarate (Tecfidera®) and fingolimod (Gilenya®). The first two drugs constitute first-line treatments, but fingolimod is licensed by the European Medicines Agency for RRMS patients with highly active or rapidly evolving severe RRMS, as described in the Summary of Product Characteristics [11].

The emergence of DMTs such as interferon-β or glatiramer acetate has considerably improved the course of RRMS. Their administration has enabled relapse frequency to be reduced and disease progression to be slowed [12, 13]. However, MS patients may still experience other undesirable effects of the disease such as spasticity, which often appears as the clinical course evolves and may affect up to 84 % of patients [14].

Spasticity arises from hyperactive stretch reflexes caused by damages in the brain and spinal cord [15], which result in increased muscle tone, spasms and/or pain that may affect patients’ movements and sphincter control [16]. As spasticity worsens, patients face increasing difficulties to perform activities of daily living and their degree of disability increases considerably. Thus, the administration of symptomatic treatment needs to be considered as a part of MS management, after considering the extent and impact of patients’ spasticity and potential exacerbating factors [16–18]. Special attention should be paid to the administration of therapies that may affect spasticity, which also includes the treatment of MS with interferon-β [16, 17]. Indeed, increased spasticity has been observed in 13–15 % of MS patients receiving interferon-β [19, 20], mainly in those with pronounced spasticity before treatment administration [21].

However, the data available on glatiramer acetate administration have not revealed any worsening of spasticity [19, 22]. Furthermore, the results of a pilot study that specifically addressed the effect of glatiramer acetate on spasticity in patients with RRMS not only supported the absence of spasticity increase but they also suggested spasticity improvements in patients previously treated with interferon-β [23]. Based on these results, we conducted a larger observational study—the Escala Study—to provide further insight into the effect of glatiramer acetate on spasticity in patients switching from interferon-β [24]. The results obtained also supported the achievement of spasticity improvements in terms of muscle tone, spasm frequency and pain, which were apparent from the third month on glatiramer acetate and remained until at least the sixth month of treatment. These improvements could additionally lead to cost savings derived from a more reduced need for spasticity treatment, representing a substantial benefit for the National Health System and contributing to its sustainability. Nevertheless, whether such improvements really translate into cost savings still remained to be assessed.

In light of the above, we conducted a cost analysis of glatiramer acetate versus interferon-β in patients with RRMS and spasticity within the context of the Escala Study and from the perspective of the National Health System of Spain.

## Methods

### Model description

This cost analysis encompassed data from the observational Escala Study [24], which included patients with RRMS recruited from 27 Spanish hospitals. Patients were between 18 and 60 years of age, had confirmed spasticity and MS treatment switched from interferon-β to glatiramer acetate (Copaxone®, Teva Pharmaceuticals Ltd., London, United Kingdom), which must have been administered for at least 24 weeks under clinical practice conditions. Investigators obtained all the study data from direct review of patients’ medical charts, including information on patients’ clinical follow-up and spasticity assessments conducted every three months after starting glatiramer acetate. Patient data were collected from September 2009 to January 2011. A total of 75 patients were recruited, seven of whom were screening failures; 68 patients comprised the evaluable population. The main reasons for switching from interferon-β were adverse reactions (55.7 %) and lack of response (40.5 %). Information on the number of MS relapses was collected, patients’ disability was assessed according to the Kurtzke Expanded Disability Status Scale (EDSS), and spasticity was evaluated according to the following specific scales: Penn Spasm Frequency Scale (PSFS), Modified Ashworth Scale (MAS), Adductor Tone Rating Scale (ATRS) and Global Pain Score (GPS); these latter scales were selected to provide an overall assessment of spasticity and spasticity-related signs such as spasms, increased muscle tone and pain. The study was conducted in accordance with the World Medical Association Declaration of Helsinki, all its amendments and national regulations. It was also approved by the ethics committees and all patients gave their written informed consent.

Since the Escala Study showed spasticity improvements after switching from interferon-β to glatiramer acetate in all previously-mentioned spasticity scales [24], a cost-comparison analysis of both therapies was conducted taking into account the potential impact of such improvements. In this analysis, patients were their own control as the cumulative costs prior to starting glatiramer acetate (interferon-β period) were compared to the subsequent six months on glatiramer acetate (glatiramer acetate period).

### Perspective, time horizon and discount rate

The analysis was carried out following the recommendations for conducting pharmacoeconomic studies and from the Spanish National Health System perspective [25]. The analysis assumed a time horizon equal to the follow-up period in the Escala Study, and was thus set to 6 months. The calculations were performed without applying a discount rate to costs, except for the sensitivity analysis.

### Resource and cost estimation

Resources used during interferon-β and glatiramer acetate periods were identified, measured and quantified according to data included in the Escala Study database. We obtained data on the use of DMTs (administration doses), spasticity treatment (different medicines, doses, and health care) and relapse management (treatments and doses). A standard price for each identified resource was assigned, taking into account the costs associated with MS treatment, spasticity treatment and relapse management (Table 1).

**Table 1:** Unit costs

Treatment	Units/Dosage forms	Cost/Unit (2014 ex-factory price)	Cost/Unit −7.5%^a^ (2014 ex-factory price)
Disease modifying treatment			
Glatiramer acetate	20 mg/ml, 28 prefilled syringes, 1 ml	781.25€	722.55€
IFN-β 1a sc	44 μcg/0.5 ml, 4 cartridges, 1.5 ml	1,167.21€	1,079.76€
IFN-β 1a im	30 μcg, 4 prefilled syringes, 0.5 ml	835.82€	773.13€
IFN-β 1b sc	250 μcg/ml, 15 powder and solvent for injection, 1.2 ml	865.00€	800.13€
Spasticity treatment			
Baclofen	10 mg, 1 vial, 5 ml	19.83€	NA†
Clonazepam	60 tablets, 2 mg	2.00€	
Diazepam	100 tablets, 5/10 mg	1.30€	
Lorazepam	20 tablets, 5 mg	2.00€	
Tetrazepam	30 tablets, 50 mg	2.83€	
Tizanidine	30 tablets, 2 mg	2.53€	
Gabapentin	90 capsules, 300 mg	5.93€	
Botulinum toxin	1 vial, 100 IU	159.12€	147.18€
Relapses cost/patient (direct and indirect costs included)	One episode	2,609.00€	NA

^a^The cost analysis is expressed in 2014 euros (€) and a discount of 7.5 % on treatment prices was applied as stipulated in Spanish law (Royal Decree 8/2010); †A price discount of 7.5 % was not applied since these are generic drugs

*im* intramuscular, *NA* not applicable, *sc* subcutaneous

#### Costs of multiple sclerosis treatment

The costs of interferon-β and glatiramer acetate were estimated according to their administration doses and unit costs, and presented as ex-factory prices; missing doses were not considered for the total costs of treatments. Drug list prices were taken from the Spanish medicines database [26] and a 7.5 % discount was applied to drug prices as stipulated by Spanish law [27].

#### Costs of spasticity treatment

Costs of spasmolytic drugs were estimated according to their administration doses and respective unit costs, and presented as ex-factory prices. Drug list prices were also taken from the Spanish medicines database [26] and a 7.5 % discount was applied—when needed—to drug prices as stipulated by Spanish law [27]; no price discount was needed for generic drugs.

#### Costs of relapse management

Costs associated with the management of relapses were estimated according to previously published relapse-related costs; it included direct treatment and informal care resource development using a costs-of-illness method [28].

### Cost-analysis

A cost-analysis was conducted taking into account costs associated with MS treatment, spasticity and relapse management, and changes in the PSFS, MAS, ATRS, GPS and EDSS as effectiveness variables. Data on these scales was retrieved from the Escala Study database. We programmed a cost model using Microsoft Excel 2013 (Microsoft Corporation, Redmond, Washington).

### Sensitivity analysis

A sensitivity analysis was conducted to verify the robustness of the model, which included two univariate analyses that assessed the impact of parameters with the most uncertainty: 1) concomitant and spasticity treatments, and 2) annual discount rates for future costs. The former included the calculation of the economic impact when considering no concomitant or spasticity treatment, without taking into account adverse-event-related costs; the latter included the calculation of costs when applying annual discount rates of 3 % and 5 %.

## Results

### Base case analysis

The Escala Study included 68 evaluable patients with RRMS and spasticity who had switched from interferon-β to glatiramer acetate. A summary of patient characteristics is given in Table 2. All these patients were considered for the costs analyses presented in this article. We selected the Escala Study because it was the only study about this topic that had been performed in Spain; it included information from the real-life clinical setting, without any intervention, and we could therefore take advance of that.

**Table 2:** Summary of patient characteristics at starting glatiramer acetate (*N* = 68)

Characteristics	Value
Age (years), mean ± SD	41.7 ± 9.5
Female, *n* (%)	48 (70.6)
Caucasian, *n* (%)	66 (97.1)
Multiple sclerosis duration (years), mean ± SD^a^	7.6 ± 5.7
Previous interferon-β for multiple sclerosis, *n* (%):	
IFN-β 1a sc	29 (42.6)
IFN-β 1a im	28 (41.2)
IFN-β 1b	22 (32.4)
Number of relapses from multiple sclerosis diagnosis, mean ± SD†	4.1 ± 3.4
EDSS score, mean ± SD	3.2 ± 4.1

^a^Missing data, *n* = 5; †Missing data, *n* = 2

*EDSS* Expanded Disability Status Scale, *im* intramuscular, *sc* subcutaneous, *SD* standard deviation

The management of RRMS, spasticity and relapses accounted for a 6-month cost per patient of 7,078.02€ when using interferon-β and 4,671.31€ when using glatiramer acetate (Table 3). Switching from interferon-β to glatiramer acetate therefore represented a cost saving of 2,406.72€ per patient in favour of glatiramer acetate that resulted from savings in treatment costs, relapse management and spasticity treatment of 1,890.02€, 430.48€ and 86.21€, respectively (Table **3**).

**Table 3:** Results of cost analyses of glatiramer acetate and interferon-β

Costs/patient	Interferon-β	Glatiramer acetate	Cost difference (interferon-β versus glatiramer acetate)
Disease modifying treatment	6.225.96€	4,335.94€	1,890.02€
Spasticity treatment	147.63€	61.42€	86.21€
Relapse management	704.43€	273.95€	430.48€
Total cost/patient	7,078.02€	4,671.31€	2,406.71€

The cost analyses are expressed in 2014 euros (€)

**Fig. 1:**
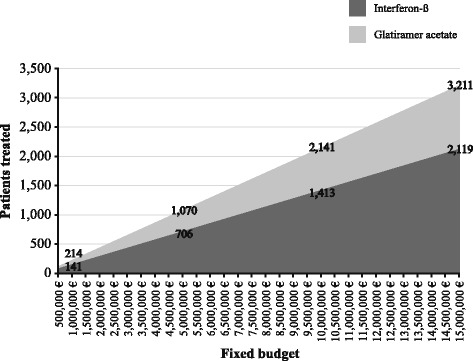
Patients treated with a fixed budget

**Fig. 2:**
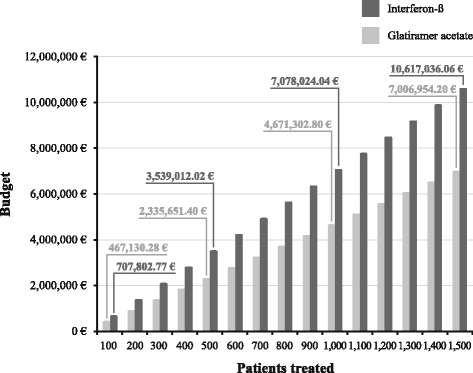
Budget depending on the number of patients treated

The ratio of the costs during interferon-β was 1.5 times the costs during glatiramer acetate, which means that a fixed budget of 5,000,000€ would enable 1,070 patients to be treated with glatiramer acetate and only 706 patients with interferon-β (Fig. 1). In addition, treating 1,000 patients with glatiramer acetate would cost 4,671,302.80€, while this figure would rise to 7,078,024.04€ if they were treated with interferon-β (Fig. 2).

### Sensitivity analysis

Results of sensitivity analysis are presented in Table 4. The assumption of no concomitant/spasticity treatment yielded overall 6-month costs of 6,225.96€ and 4,335.94€ during interferon-β and glatiramer acetate treatments, respectively. When a discount rate of 3 % was applied, the costs during interferon-β were 6,871.86€ and those during glatiramer acetate were 4,535.25€. After applying a discount rate of 5 %, these costs were reduced to 6,740.97€ for interferon-β and 4,448.87€ for glatiramer acetate.

**Table 4:** Results of sensitivity analysis

Cost/patient	Interferon-β	Glatiramer acetate	Cost difference (interferon-β versus glatiramer acetate)
Base case	7,078.02€	4,671.31€	2,406.71€
When considering no concomitant/spasticity treatment	6,225.96€	4,335.94€	1,890.02€
When applying an annual discount rate of 3 %	6,871.86€	4,535.25€	2,336.61€
When applying an annual discount rate of 5 %	6,740.97€	4,448.87€	2,292.10€

The cost analyses are expressed in 2014 euros (€)

## Discussion

This study supports that the treatment of RRMS with glatiramer acetate entails cost savings derived from reduced expenses in therapy cost, relapse management and spasticity improvement when compared to interferon-β. Indeed, our study showed an overall 6-month cost per patient of 4,671.31€ during glatiramer acetate treatment and a cost saving for the Spanish National Health System of 2,406.71€ when compared to interferon-β, which resulted from savings of 1,890.02€ in disease modifying therapies, 430.48€ in relapse management and 86.21€ in spasticity treatment. These cost savings represent additional benefits to the spasticity improvements that were previously observed in terms of PSFS, MAS, ATRS and GPS scores after switching from interferon-β to glatiramer acetate [24]. Added to the underlying pathophysiology of spasticity, certain external factors and drugs such as interferon may exacerbate the existing condition; hence, their awareness is crucial as part of an effective management of spasticity. Furthermore, the evidence for the effectiveness of glatiramer acetate in preventing spasticity in naïve patients and in those switching from interferon should not be ignored [29].

Apart from the evidence on the clinical benefits of therapies, national health services also need to take into account their respective budget impact to efficiently allocate the recourses needed for disease management [30, 31]. Previous economic evaluations carried out in Spain have reported that the annual costs per patient of MS during glatiramer acetate may range from 9,439.42€ to 32,250.996€ [32–36], while those during interferon-β vary from 9,955.74€ to 37,493.45€ [32, 34–37]. In addition, cost-comparison analyses conducted in our country have also pointed out that glatiramer acetate represents a less costly strategy for managing RRMS that may led to substantial cost savings for the Spanish National Health System when compared to interferon-β [32, 34–36]. Deterministic results showed that the expected annual cost per patient was lower when treated with glatiramer acetate (13,843€) compared with interferon (15,589€), and the combined treatment with both interferon and glatiramer acetate (21,539€) [36]. The annual number of relapses was lower in the glatiramer acetate cohort with 3.81 versus 4.18 in the interferon cohort and 4.08 in the cohort treated with interferon plus glatiramer acetate. Results from probabilistic sensitivity analysis showed that glatiramer acetate had a higher probability of being cost-effective than the treatment with interferon or interferon plus glatiramer acetate for threshold values from 28,000€ onwards, independently of the maximum expense that the Spanish National Health System is willing to pay for avoiding relapses [36]. Glatiramer acetate was shown to be a cost-effective treatment option to prevent relapses in Spanish patients diagnosed with RRMS. When glatiramer acetate monotherapy was compared with interferon monotherapy and with interferon plus glatiramer acetate, it might be concluded that the first was the dominant strategy [36]. Even though comparison of economic evaluations from other counties is always difficult due to differences in methodology, design, patient populations and health systems, the data available supports the overall lower costs of MS management with glatiramer acetate versus interferon-β [38–45]. Recently, the large clinical cohort of RRMS patients starting a DMT of the United Kingdom Multiple Sclerosis Risk Sharing Scheme approached the long-term disability progression and cost-effectiveness of interferon-β and glatiramer acetate. The findings from this observational study supported that the effects of interferon-β and glatiramer acetate on disability in RRMS patients were maintained and cost-effective even over 6 years [46].

The lower acquisition cost of glatiramer acetate compared to interferon-β may have contributed to the lower costs reported in the previously mentioned analyses and highlights the importance of efficient management of disease modifying therapies in the economic impact on the health system. However, these evaluations did not quantify the impact of spasticity, which may entail additional costs such as those derived from the administration of spasmolytic drugs. Our results support this hypothesis as spasticity treatment during interferon-β accounted for approximately 148€ per patient and the spasticity improvement observed after switching to glatiramer acetate enabled this figure to be reduced by more than one-half.

Several analyses have reported that the costs of managing MS are mainly driven by the utilization of healthcare resources and the costs of disease modifying therapies [7, 44, 45, 47]. However, a Spanish retrospective assessment of MS patients with resistant spasticity also pointed out this factor as a contributor to overall costs of MS management, though to a lesser extent than disease modifying therapies and care provision [48]. Subsequently, another Swedish study conducted in MS patients with spasticity raised the issue of the substantial burden of spasticity on the healthcare system, social services, patients and relatives/caregivers [49]. This burden may increase over the course of MS and spasticity worsening, mainly driven by the increased need for personal assistance and patient productivity losses [49]. A recently published online survey among health care specialists in the management of MS and spasticity in the United Kingdom also revealed the high impact of spasticity on both health care resources and costs according to the different levels of spasticity [50]. The higher degrees of spasticity were associated with increased need for clinic visits, accident and emergency attendance, hospital admissions, district nurse support, home care and specialised home equipment. This use of resources was associated with additional costs that increase as spasticity worsens, with a predominant impact of increased home care, clinic visits and hospital admissions to the overall annual costs in patients with more severe spasticity. Therefore, any reduction in spasticity achieved through either early or effective management of spasticity could not only improve patients’ wellbeing but may also lead to substantial cost savings [49, 50]. Furthermore, this survey provides additional insight into the complexity of the economic implications of spasticity in MS, which need to be carefully considered by national stakeholders to ensure the sustainability of health systems.

The authors acknowledge that their economic analysis has limitations that should be considered when interpreting its findings. Although prospective determination of costs would be more appropriate for this type of studies [51], only was retrospective data available in the Escala Study; therefore, limitations derived from the availability of data should be considered. In addition, the absence of a control group should be taken into account and further controlled studies should be conducted to avoid potentially confounding factors. Although the study sample may look small, the sample size determination was based on a pilot study that evaluated the effects of glatiramer acetate on spasticity in patients with RRMS [18]. The scale with the lowest variation during the study was considered to guarantee statistical significance of the variable with the lowest change; the selected variable was the ATRS, whose mean absolute change ranged from 0.55 to 0.20, and the most conservative value (0.20) was used to calculate the sample size, together with the standard deviation of patients previously treated with interferon-β (0.69) [24]. Nonetheless, caution is advisable when interpreting the study findings and additional larger studies would be desirable to achieve confirmatory evidence on this matter. Moreover, potential cost savings derived from the reduced number of patients missing working days over glatiramer acetate—just five as result of spasticity–[24] could not be considered due to the lack of data on patients missing working days over interferon-β; however, it can be hypothesized that spasticity improvements observed during glatiramer acetate may lead to additional cost savings resulting from reduced spasticity-related work leaves. Furthermore, this analysis was conducted from the perspective of the National Health System of Spain, taking into account only direct healthcare costs and not considering indirect costs such as loss of productivity. Nevertheless, the authors believe that this analysis provides meaningful information on the management of MS patients with spasticity in clinical practice as it was based on real-life data instead of a literature review or expert opinion.

## Conclusions

The administration of glatiramer acetate for RRMS entailed cost savings after six months of treatment when compared to interferon-β in patients with spasticity, which not only resulted from its lower costs of therapy and relapse management but also from its favourable effect on reducing spasticity. The spasticity improvement observed after switching from interferon-β to glatiramer acetate may be accompanied by a cost reduction that might contribute to the sustainability of our health system. Thus, glatiramer acetate may be regarded as a more efficient alternative than interferon-β from the perspective of the National Health System of Spain. Nevertheless, our economic analysis needs to be placed within the context of the Escala Study and further larger long-term analyses are still needed to confirm its findings, as well as providing additional evidence on the impact of indirect costs and differences in health systems between counties.

## Abbreviations

ATRS: Adductor Tone Rating Scale
EDSS: Expanded Disability Status Scale
GPS: Global Pain Score
im: Intramuscular (only used in tables)
MAS: Modified Ashworth Scale
MRI: Magnetic resonance imaging
MS: Multiple sclerosis
NA: Not applicable (only used in a table)
PSFS: Penn Spasm Frequency Scale
RRMS: Relapsing-remitting multiple sclerosis
sc: Subcutaneous (only used in tables)
SD: Standard deviation (only used in a table)
